# Chronic Social Stress Leads to Reduced Gustatory Reward Salience and Effort Valuation in Mice

**DOI:** 10.3389/fnbeh.2018.00134

**Published:** 2018-07-10

**Authors:** Diana Kúkel’ová, Giorgio Bergamini, Hannes Sigrist, Erich Seifritz, Bastian Hengerer, Christopher R. Pryce

**Affiliations:** ^1^Preclinical Laboratory for Translational Research into Affective Disorders, Department of Psychiatry, Psychotherapy and Psychosomatics, Psychiatric Hospital, University of Zurich, Zurich, Switzerland; ^2^Department of Animal Physiology, Institute of Biology and Ecology, Faculty of Science, P. J. Šafárik University in Košice, Košice, Slovakia; ^3^Department of Psychiatry, Psychotherapy and Psychosomatics, Psychiatric Hospital, University of Zurich, Zurich, Switzerland; ^4^CNS Diseases Research Germany, Boehringer Ingelheim Pharma GmbH & Co. KG., Biberach, Germany

**Keywords:** social stress, RDoC, reward salience, reward effort, glucose, leptin, mouse model

## Abstract

Pathology of reward processing is a major clinical feature of stress-related neuropsychiatric disorders including depression. Several dimensions of reward processing can be impacted, including reward valuation/salience, learning, expectancy and effort valuation. To establish the causal relationships between stress, brain changes, and reward processing pathologies, valid animal models are essential. Here, we present mouse experiments investigating behavioral effects of chronic social stress (CSS) in association learning tests of gustatory reward salience and effort valuation. The reward salience test (RST) comprised Pavlovian pairing of a tone with gustatory reward. The effort valuation test (EVT) comprised operant responding for gustatory reinforcement on a progressive ratio schedule (PRS). All testing was conducted with mice at 100% baseline body weight (BBW). In one experiment, mice underwent 15-day CSS or control handling (CON) and testing was conducted using sucrose pellets. In the RST on days 16–17, CSS mice made fewer feeder responses and had a longer tone response latency, than CON mice. In a shallow EVT on days 19–20, CSS mice attained a lower final ratio than CON mice. In a second CSS experiment, mice underwent CSS or CON and testing was conducted with chocolate pellets and in the presence of standard diet (low effort/low reward). In the RST on days 16–18, CSS mice made fewer feeder responses and had a longer tone response latency, than CON mice. In a steep EVT on days 19–20, CSS and CON mice attained less pellets than in the RST, and CSS mice attained a lower final ratio than CON mice. At day 21, blood levels of glucose and the satiety adipokine leptin were similar in CSS and CON mice. Therefore, CSS leads to consistent reductions in reward salience and effort valuation in tests based on association learning. These reward pathology models are being applied to identify the underlying neurobiology and putative molecular targets for therapeutic pharmacology.

## Introduction

Stressful life events are major etiological factors for prevalent neuropsychiatric disorders, including depression. Environmental stressors that precede the onset of depression are often psychosocial and uncontrollable and, therefore, chronic (Kessler, 1997; Kendler et al., 1999; Pryce et al., 2011). Depression is heterogeneous with respect to symptomatology, as indeed are other stress-related neuropsychiatric disorders such as schizophrenia. A core symptom of depression is described as markedly reduced interest or pleasure in (almost) all activities most of the day nearly every day (Dichter et al., 2010). Impaired processing of rewarding events/stimuli also characterizes negative symptoms in schizophrenia (Dichter et al., 2010; Hartmann et al., 2015). The research domain criteria (RDoC) framework places focus on the study and understanding of specific psychological processes, the dysfunction of which is relevant to one or more neuropsychiatric disorders, i.e., transdiagnostic (Cuthbert and Insel, 2013). “Positive valence systems” is the RDoC term for constructs underlying reward-stimulus processing, and examples include: valuation of the salience of a prospective reward (anticipation); learning the association between neutral stimuli and reward (Pavlovian learning), or behavioral actions and reward (operant learning); reward expectancy triggered by stimuli associated with reward; effortful motivation to obtain reward^1^.

Automated behavioral tests of such processes are essential for their quantitative study in psychiatric patients compared with healthy control probands, including assessment of the relationship with psychometric measures. Tests of relevance to reward processes that have been applied in depression research to date are based on stimulus discrimination or stimulus choice. For example, Pizzagalli et al. (2005) presented non-clinical subjects with two visual stimuli, one per trial, and correct identification of one stimulus was reinforced-using money-more frequently than correct identification of the other stimulus; such an asymmetric reinforcement ratio leads to a response bias for the more frequently reinforced stimulus. Subjects who scored relatively highly on the self-report psychometric scale, the Beck Depression Inventory, developed a lower reward bias than did subjects with a relatively low depression score. A low response bias for reward is consistent with reduction in one or more of reward valuation, reward learning and reward expectancy, and further tests could be applied to establish which of these is primarily responsible. Treadway et al. (2012) presented depressed and healthy subjects with a choice between high-effort/high-reward and low-effort/low-reward trials, where reward was monetary and, across trials, the size and probability of reward (both low- and high-reward) were variable but predictable. Relative to controls, depressed patients made less high-effort/high-reward choices and were less responsive to size and probability of reward. These effects are clearly consistent with reduced effortful motivation in depression, and also suggest deficient reward valuation and reward learning. In a monetary incentive delay task conducted with functional magnetic resonance imaging (fMRI), one visual stimulus predicts reward (money) and another predicts no reward. In the interval between stimulus response and reward feedback, subjects exhibit increased activity in the ventral striatum/nucleus accumbens. Relative to controls, depressed and schizophrenic patients exhibited lower activation of ventral striatum (Arrondo et al., 2015), and the magnitude of activation was inversely correlated with the Beck Depression Inventory score (Hagele et al., 2015). The nucleus accumbens receives inputs from dopamine neurons in the ventral tegmental area, a major pathway in the mesolimbic dopamine neural circuit of reward processing (Pizzagalli, 2014). Therefore, behavioral tests have identified quantitative deficits in the reward-processing dimensions of approach motivation and possibly also learning, in depressed patients. Interestingly, consummatory pleasure, as measured by the Sweet taste test, is not reduced in depression (Dichter et al., 2010; Treadway, 2016).

Animal models of stress-induced deficits in reward processing analogous to these pathological dimensions in depression are challenging to develop but essential: they enable the causal study of the pathways via which environmental stressors lead to pathophysiological states in reward neurocircuitry and processing, and thereby allow for investigation of novel therapeutic strategies. Given that psychosocial stressors are the major “environmental pathogens” for human emotional disorders (Caspi and Moffitt, 2006), animal models based on manipulations of the social environment would be expected to have particularly high etiological validity (Markou et al., 2009; Pryce and Seifritz, 2011). Examples of social stressors that have been deployed in conjunction with tests of reward include early-life stress in the form of repeated infant-parent separation and chronic adulthood stress in the form of intruder-resident confrontation. In monkeys, for example the common marmoset, early life stress resulted in impaired reversal learning and effort valuation (Pryce et al., 2004), whilst direct neurotoxic depletion of orbitofrontal cortex dopamine also reduced reward sensitivity in a discrimination learning paradigm (Clarke et al., 2014). In rodents, most stress-reward modeling has been conducted in rats to-date. Emphasis has been on the sweet preference test (SPT), in which consumption of sucrose/saccharin relative to water is measured. The initial demonstration (Willner et al., 1987) that chronic unpredictable mild stress (CUMS, a combination of physical and psychosocial stressors repeated over several weeks) led to a reduced sucrose preference that could be restored by antidepressant administration has resulted in a huge number of studies (>1000) with this model (Willner, 2017). However, the SPT is not a test for the dimensions that underlie deficient reward interest in depression, but rather a test for sustained responsiveness during consummatory behavior (Cuthbert and Insel, 2013). Tests of reward bias with analogy to the human test described above (Pizzagalli et al., 2005) have been developed for rats (Der-Avakian et al., 2013, 2016, 2017). In one test version, rats are trained to discriminate between two tone stimuli by pressing the specific lever associated with each tone. In the test phase, they are then presented with ambiguous tones, one of which is more frequently rewarded than the other. Rats exposed to 3 days of social stress had a reduced response bias toward the more frequently rewarded tone (Der-Avakian et al., 2017). In another test version, low or high amounts of food reward are each associated with specific substrates (conditioned stimuli); following reward-substrate learning, rats are presented with a substrate choice test with reward now randomized across substrates. Rats treated chronically with the pro-inflammatory cytokine interferon-α developed a low reward bias relative to controls, but retained a normal sucrose preference in the SPT (Stuart et al., 2017). Choice tests for high effort/reward vs. low effort/reward, analogous to the test used to detect effort valuation deficits in depression (Treadway et al., 2012), have also been developed in rats. A major example is concurrent lever pressing for sucrose vs. eating of “free” standard chow; lever pressing is reduced by dopamine antagonists or nucleus accumbens dopamine depletion (Salamone et al., 2007, 2016a,b). Effort valuation of sucrose in the absence of an alternative low reward has been assessed using operant lever pressing on reinforcement schedules, most notably the progressive ratio schedule (PRS; Salomons et al., 2007). Rats exposed to early life stress exhibited, in adulthood, less effort to earn sucrose in a PRS test, a deficit reversed by repeated antidepressant (fluoxetine) treatment (Leventopoulos et al., 2009).

In male mice, we have demonstrated a number of effects on reward processing of a 15-day resident-intruder paradigm that we refer to as chronic social stress (CSS). The paradigm is based on male-male aggression and can therefore not be applied to females; given the relatively high prevalence of depression in women, this is an important caveat. Nonetheless, in male mice, CSS leads, relative to control mice, to reduced operant responding in a time-restricted fixed ratio 1 (FR1) test and a PRS test (Bergamini et al., 2016a, 2018), as well as to fewer spontaneous episodes of approach and FR1 responding to access saccharin or water in a complex home cage environment (Bergamini et al., 2016a). CSS is without effect in a saccharin preference test (Bergamini et al., 2018). Such behavioral effects are consistent with stress-induced deficits in reward salience and effort valuation. Furthermore, in a two-way spatial reversal learning test, CSS mice exhibited less reward-stay responses and made more errors to reversal, consistent with deficits in one or more of reward valuation, learning or expectancy (Bergamini et al., 2016a). Also under two-way spatial learning conditions, CSS mice required more trials to learn that a previously non-rewarded stimulus was now rewarded, consistent with impaired reward learning (Bergamini et al., 2018). Importantly, this latter effect of CSS and that of attenuated PRS responding are also induced by pharmacological nucleus accumbens dopamine depletion (Bergamini et al., 2016b). Furthermore, CSS leads to attenuated mesolimbic dopamine pathway functioning (Bergamini et al., 2018). Building on the findings with this mouse model to-date, the aims of the present study were to investigate CSS effects on gustatory reward valuation and reward learning in a test of Pavlovian conditioning and on reward effort valuation using an operant PRS. We hypothesized that CSS would lead to reduced salience of gustatory reward, particularly in terms of impaired acquisition of the predictive association of a tone with reward availability, and to an additional reduction in reward valuation under conditions of sustained effort to obtain the reward.

## Materials and Methods

### Animals and Housing

**Figure 1:**
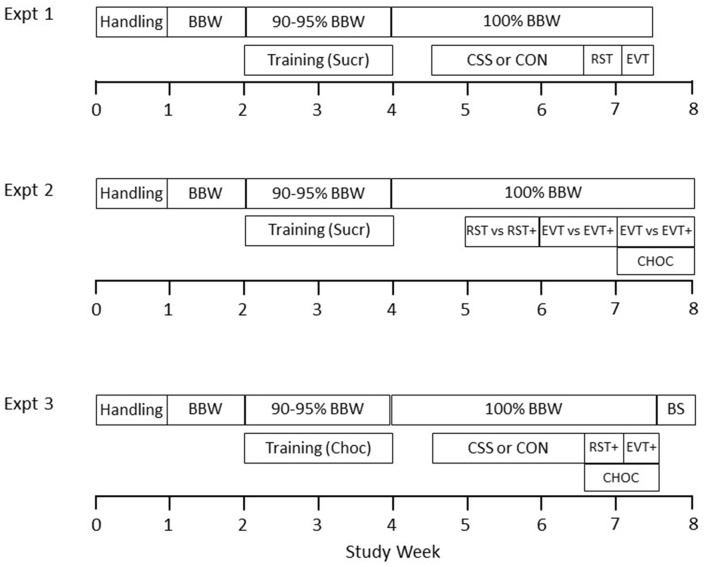
Designs of the three experiments conducted in this study. First, in each experiment, naive mice were handled and then body weight (BW) and chow intake were measured on each day for 7 days, to calculate baselines for body weight (BBW) and chow intake. Second, mice were chow-deprived to reduce BW to 90%–95% BBW, and then placed each day into the behavioral apparatus for training of collection of sweet pellets from the feeder and operant nose poking to initiate pellet delivery. Pellets were either sucrose (Sucr) or chocolate (Choc). Experiment 1: mice were exposed to chronic social stress (CSS) or control handling (CON) handling for 15 days, followed by two daily sessions in the reward salience test (RST) and two daily sessions in the effort valuation test (EVT), with Sucr pellet reward. Throughout this period, mice were fed sufficient daily chow to maintain them at 100% BBW. Experiment 2: mice were given three daily sessions in the RST either with (RST+) or without a chow pellet in the test chamber and with Sucr pellet reward. They were then given two sessions at a 2–3-day interval in the EVT with Sucr pellet reward, one with (EVT+) and one without a chow pellet (order counterbalanced), and finally two sessions at a 2–3-day interval in the EVT with Choc pellet reward, one with and one without a chow pellet (order counterbalanced). Throughout this period, mice were fed sufficient daily chow to maintain them at 100% BBW. Experiment 3: mice were exposed to CSS or CON handling for 15 days, followed by three daily sessions in the RST with a chow pellet and two daily sessions in the EVT with a chow pellet, and Choc pellet reward. Throughout this period, mice were fed sufficient daily chow to maintain them at 100% BBW. On the day following completion of behavioral testing, trunk blood sampling (BS) was conducted for plasma glucose and leptin determinations.

The study was conducted with C57BL/6J (BL/6J) male mice bred in-house. Breeding pairs each contributed 1–2 offspring to one or both treatment groups, i.e., CSS, control. Mice were weaned into littermate-pairs at age 3 weeks and remained in these pairs throughout the experiment or until the onset of CSS. Mice in Experiment 1 were transferred from a non-reversed light-dark cycle to a reversed cycle at age 5 weeks, whilst in Experiments 2 and 3 mice were born and remained in reversed cycle conditions. At study onset, mice were aged 12–13, 10–11 and 10–11 weeks in Experiments 1, 2 and 3, respectively (Figure 1). Mice were maintained in individually-ventilated type 2L cages containing wood chips, tissue bedding and a sleeping igloo. The temperature was set at 20–22°C and humidity at 50%–60%, and illumination was on a reversed 12:12 h light-dark cycle (white lights off at 07:00–19:00 h). Experimental procedures were conducted during the dark phase, at 08:00–16:00 h. In the home cage, standard chow diet (Complete pellet, Provimi, Kliba AG, Kaiseraugst, Switzerland) was available as detailed below, and water was available continuously. For CSS, the resident mice were ex-breeder males of the CD-1 strain (Janvier Labs, Saint-Berhevin, France) aged 8 months, weighed 37–56 g, and caged singly. This study was carried out in accordance with the recommendations of the Animal Protection Act, Switzerland. The protocol was approved and permit issued (ZH 149/2015) by the Cantonal Veterinary Office, Zurich, Switzerland. All efforts were made to minimize the number of mice studied and any unnecessary stress to those mice that were studied.

### Experimental Design

Each of three experiments was conducted with a different, naive mouse cohort (Figure 1). Experiments began with mouse handling on each of 5 days. During the next week, baseline body weight (BBW) and food consumption were measured daily. Mice were then food deprived to reduce them to 90%–95% BBW and underwent training with sucrose or chocolate pellet reinforcement during 2 weeks. Post-training, they were then provided with sufficient daily chow to maintain them at 100% BBW for the remainder of the experiment. In Experiment 1, CSS (*N* = 19) and control handling (CON, *N* = 18) were conducted for 15 days, followed by behavioral testing in the reward salience test (RST) and the shallow effort valuation test (EVT) with sucrose pellets. In Experiment 2, mice (*N* = 23) were tested in the RST with sucrose pellets, with or without normal chow in the test cage, the steep EVT with sucrose pellets with or without normal chow, and the steep EVT with chocolate pellets with or without normal chow. In Experiment 3, CSS (*N* = 10) and CON (*N* = 10) were conducted for 15 days, followed by behavioral testing in the RST and the steep EVT, with chocolate pellets. On the day after the end of behavioral testing, blood collection was conducted from these mice for plasma glucose and leptin determination.

### Chronic Social Stress

Mice were allocated to CSS and CON groups by counterbalancing for BBW and number of pellets obtained in the final training sessions (see below). CSS was conducted according to the resident-intruder protocol described in detail elsewhere (Azzinnari et al., 2014). Briefly, unfamiliar, aggressive, ex-breeder CD-1 mice were caged singly and with a transparent, perforated plastic divider separating the cage into two equal compartments. On each of 15 days the BL/6J CSS mouse was placed in the same compartment as the CD-1 mouse for a total of 60 s physical attack or 10 min maximum. After the physical attack, the CSS mouse remained in the same compartment and the CD-1 mouse was transferred to the opposite compartment for 24 h, during which the two mice had visual, olfactory, auditory and limited tactile contact. The CSS × CD-1 mouse pairings were rotated so that each CSS mouse was confronted with a novel CD-1 mouse each day. To prevent bite wounds the lower incisors of CD-1 mice were trimmed every 3rd day. From day 15 and throughout behavioral testing, each CSS mouse remained in the same divided cage with the same CD-1 mouse without further attacks. As expected, all CSS mice displayed submissive behavior and vocalization during the 10 min periods of proximate contact, and all CD-1 mice attacked. The mean duration of daily attack received by CSS mice was 45–50 s (Azzinnari et al., 2014). Control (CON) mice remained in their littermate pairs during the dark period and the littermates were separated by a divider during the light period; this was done to increase the similarity of the thermal and metabolic demands on CSS and CON mice during this primarily inactive period (Gordon et al., 1998).

### Behavioral Tests

#### Controlled Feeding and Body Weight

Body weight (BW) and free-feeding chow intake per littermate pair were measured every day for 1 week and mean values provided baselines for each mouse; for food intake, weight eaten per mouse was calculated by assuming chow consumption was proportional to BW. Beginning the following week, mice were chow restricted to 90%–95% of baseline BW (BBW) to ensure adequate motivation during operant training with sweet pellet reinforcement. After completion of training, for 3 days prior to and throughout CSS/CON and behavioral testing in Experiments 1 and 3, and for 1 week prior to and throughout testing in Experiment 2, mice were provided with sufficient standard diet to return to and maintain 100% BBW. As described previously (Bergamini et al., 2016a, 2018), CSS mice require more standard diet per day to maintain 100% BBW than do CON mice. During the behavioral testing period, the daily chow was provided in the home cage 2–3 h after operant training/testing, and all chow had been consumed prior to the time of testing on each day.

#### Test Apparatus

Behavioral testing was conducted in an infra-red illuminated room adjacent to the mouse holding room, between 09:00 h and 13:00 h. Modular operant chambers (TSE Systems) were used with inner dimensions of 20 × 17 × 18 cm and a house light provided 10 lux illumination. In the middle of one side wall, a feeder port (∅ = 20 mm × depth = 35 mm) was located into which food pellets were delivered from a pellet dispenser. Each nose poke by the mouse into the port was detected via infra-red beam. A tone stimulus could be presented via a speaker located above the feeder port. An operant nose-poke port could be inserted to the side of the feeder port (∅ = 20 mm × depth = 30 mm); a white lamp set into the recess of this port was illuminated to indicate it was active, and nose pokes were detected via an infra-red beam. The center-to-center distance between the nose-poke port and the central feeder was 55 mm. Four such chambers, each placed within an attenuation chamber, were run in parallel by the control PC and interface. The chamber floor and walls were wiped with 70% ethanol between each run. Further details are provided in Ineichen et al. (2012).

#### Training

Training sessions were conducted on consecutive days and had a maximum duration of 30 min. First, without the operant nose-poke port, mice were trained that sucrose pellets were available in the feeder port. In the first session 15 pellets were placed in the feeder port and one further pellet was delivered automatically each 45 s; in subsequent sessions only one pellet was placed in the feeder port at session onset and mice were required to eat ≥30 pellets in one session; 3–7 sessions were required. At the next stage, mice were required to nose poke once into the feeder to trigger each pellet delivery and learning criterion was two consecutive sessions with ≥30 pellets retrieved and eaten; mice required 2–7 sessions. Then the nose-poke port was introduced and one nose-poke (FR1) into the illuminated port was required to extinguish the light and trigger pellet delivery; pellet retrieval was followed by a 5 s time out and the nose-poke port was then illuminated again. In the first three sessions, 5, 3 and 1 pellets, respectively, were placed in the nose-poke port. Mice were required to earn and eat ≥30 pellets in two consecutive sessions; 3–6 sessions were needed. In Experiments 1 and 2 mice were trained with sucrose pellets (14 mg Dustless Precision Pellets, Bio-Serv), and in Experiment 3 with chocolate-flavor sucrose pellets (20 mg Dustless Precision Pellets, Bio-Serv).

Mice required 8–15 days to complete the three training stages. They were then given sufficient standard diet each day to restore 100% BBW. In Experiments 1 and 3, after 3 days the CSS/CON procedure was then started followed by behavioral testing, and in Experiment 2 behavioral testing was started 7 days after training completion. The number of pellets earned in the final training session and the BBW were used to counterbalance allocation of mice to CSS and CON groups.

#### Reward Salience Test (RST)

The chamber contained the feeder port and no operant nose-poke port. The session was initiated by presenting a novel tone at 6.5 kHz and 80 dB; the tone had a maximum duration of 30 s and one response into the feeder triggered immediate pellet delivery and tone termination after 1 s. The intervals between consecutive CSs were variable and 50 ± 30 s (inter-trial interval, ITI). Feeder responses during the ITI were counted but were without consequence. Therefore, the tone was a conditioned stimulus (CS) that predicted pellet availability in the feeder. The maximum number of trials per session was 50 and the maximum session duration was 60 min. Two RST sessions were run on consecutive days in Experiment 1 and three such sessions in Experiments 2 and 3. Outputs of interest were number of pellets obtained, latency to respond during CS, and number of responses during ITI. For analysis, the following measures were calculated for the first 30 CS trials per session: pellets obtained; mean CS response latency; mean ITI response latency (ITI duration/ITI responses); CS/ITI reciprocal ratio (1/(CS response latency/ITI response latency)) i.e., if CS response latency < mean ITI response latency, then ratio > 1. Sucrose pellets were used in Experiments 1 and 2 and chocolate pellets in Experiment 3. In Experiment 2, half of the mice were tested without and the other half with a normal chow pellet (3 g) placed on the floor of the chamber. In Experiment 3, all mice were tested with a chow pellet available. The weight of chow pellet eaten was calculated.

#### Effort Valuation Test (EVT)

The EVT began on the day following completion of the RST. The chamber now contained the operant nose-poke port and feeder port. The session was initiated with illumination of the nose-poke port, and one nose poke resulted in a 1 s tone (6.5 kHz, 80 dB) and delivery of a pellet into the feeder; pellet retrieval was followed by a 5 s time out and then the nose-poke port was illuminated again. In Experiment 1, the following, shallow PRS of reinforcement was used: trials 1–8 = 1 response/trial, 9–16 = 3 responses/trial, 17–24 = 5, 25–32 = 7, 33–40 = 9, and so on. In Experiments 2 and 3, the following, steep PRS was used: trials 1–5 = 1, 6–10 = 5, 11–15 = 9, 16–20 = 13, 21–25 = 17. The session duration was 45 min. In previous studies (Ineichen et al., 2012; Bergamini et al., 2016a) a break point was used, defined as no response at either one or both of the nose-poke and feeder ports for 600 s; however, since this occurred only rarely we did not use a break point in this study. Outputs/measures of interest were: number of operant responses, number of pellets earned, final ratio attained, total number of feeder responses and feeder response latency, and weight of chow eaten. In Experiment 1, the EVT was run on two consecutive days and sucrose pellets were used. In Experiment 2, all mice were tested in four tests with 2–3-day intervals between consecutive tests: the first two tests were with sucrose pellets and mice were counterbalanced with respect to being tested with a normal chow pellet in the chamber for either the first or second test; these mice were then introduced to chocolate pellets in their home cage on 2 days, and in the following days the EVT ± chow pellet procedure was repeated with chocolate pellets. In Experiment 3, the EVT was run on two consecutive days with chocolate pellets and with a chow pellet in the chamber.

### Plasma Glucose and Leptin Determination

In Experiment 3, on the day after completion of behavioral testing, mice were decapitated and trunk blood was collected into EDTA-coated tubes (Microvette 500 K3E, Sarstedt) and placed on ice. Blood samples were centrifuged at 3000 rpm for 15 min at 4°C and plasma aliquots were transferred to Protein Lobind tubes (Eppendorf) and stored at −80°C. Plasma glucose was determined using a hand-held glucose meter (Accu-Chek). Plasma leptin was determined using an ELISA kit (Mouse Leptin EZML-82K, Merck Millipore) according to the manufacturer’s protocol: standards (0.23–30 ng/mL) were run in triplicate and samples in duplicate, and leptin concentrations of low and high quality controls provided with the kit were within the expected range.

### Data Analysis

For the reward valuation test, each daily session comprising CS 50 trials, data analysis focused on the first 30 CS trials per session i.e., trials 1–30 in session 1, trials 51–80 in session 2 and trials 101–130 in session 3. Mixed factorial analysis of variance (ANOVA) was used: in Experiments 1 and 3, for each output measure there was a between-subject factor of group (CSS, CON) and within-subject factor of trials; in Experiment 2, there was a between-subject factor of chow pellet (with, without) and within-subject factor of trials. For the EVT, in Experiments 1 and 3 the effects of CSS on behavior were analyzed separately for the two daily sessions, using unpaired *t*-tests. For Experiment 2, a 2 × 2 repeated measures ANOVA was used with within-subject factors of reward (sucrose, chocolate) and chow pellet (with, without). In each experiment, findings in the EVT were similar for both daily sessions, and data are reported for the second session specifically. Significant interaction effects were analyzed using *post hoc* pairwise comparisons with Bonferroni correction. Statistical significance was accepted at *p*-value ≤ 0.05. Effect size values were calculated as partial eta squared (*η*^2^) for ANOVA and as Cohen’s *d* for *t*-test. Data are presented as means and where an estimate of variance is given this is 1 standard deviation (SD).

## Results

### Experiment 1

**Figure 2:**
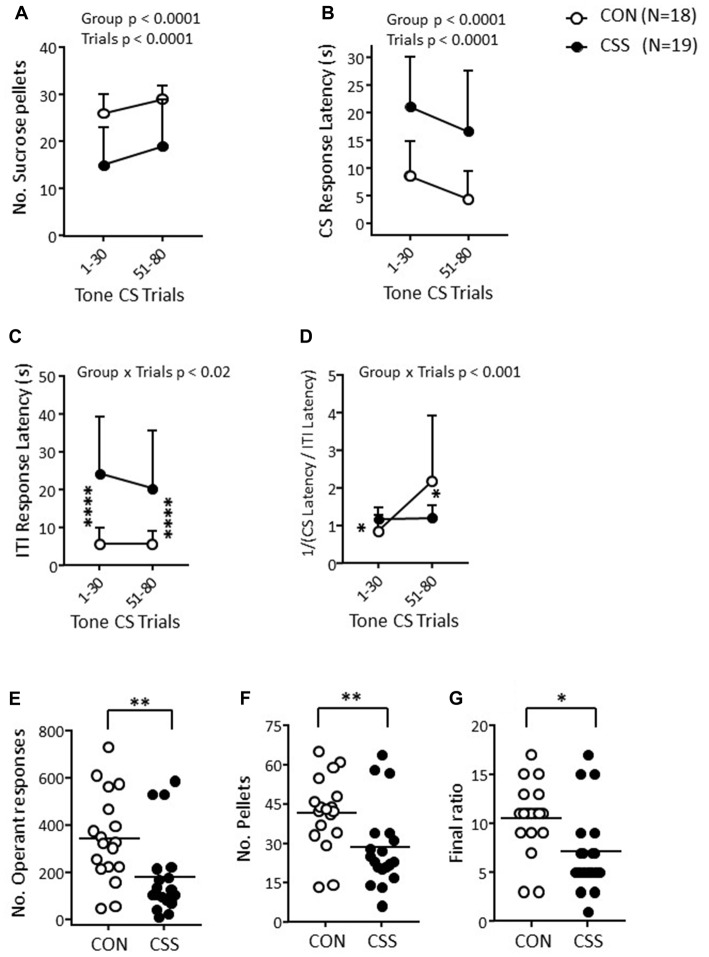
Effects of CSS on days 1–15 on reward-directed behavior in Experiment 1. **(A–D)** Scores for the first 30 trials per session in the RST on days 16–17: **(A)** Number of sucrose pellets obtained. **(B)** Mean response latency to a tone conditioned stimulus (CS; maximum duration 30 s) associated with pellet availability. **(C)** Mean response latency in the inter-trial intervals (ITI, 50 ± 30 s) between successive CS. **(D)** Mean CS/ITI reciprocal ratio. Values are overall mean ± SD. Significant statistical effects indicated were obtained with mixed factorial analysis of variance (ANOVA); *p < 0.05, ****p < 0.0001, in *post hoc* tests of single sessions following Group η Trial interaction. **(E–F)** Scores for the EVT on day 19, using a shallow progressive ratio schedule (PRS). **(E)** Number of operant responses. **(F)** Number of sucrose pellets earned. **(G)** Final ratio attained. Graphs are scatter plots and mean values per group. Significant statistical effects indicated were obtained with unpaired *t*-tests; *p < 0.05, **p < 0.01.

During the 15-day period of environmental manipulation, CON and CSS mice were maintained at 101.4% ± 2.0% and 101.2% ± 2.2% BBW, respectively. This required providing the baseline amount of daily chow to CON mice and 123% of baseline daily chow to CSS mice (Table 1). During the subsequent period of behavioral testing, both groups were again maintained at BBW, and this required 95% of baseline daily chow in CON mice and 115% thereof in CSS mice (Table 1). In the RST, CSS mice obtained fewer pellets (Figure 2A) than CON mice across the first 30 trials of each of the two sessions (Group main effect: *F*_(1,35)_ = 23.17, *p* < 0.0001, *η*^2^ = 0.40). Both CON and CSS mice increased the number of sucrose pellets obtained from session 1 (trials 1–30) to session 2 (trials 51–80; Trials main effect: *F*_(1,35)_ = 22.19, *p* < 0.0001, *η*^2^ = 0.39). The CS response latency (Figure 2B) was longer in CSS than CON mice (*F*_(1,35)_ = 23.83, *p* < 0.0001, *η*^2^ = 0.41), whilst in both CON and CSS mice it decreased similarly across sessions (*F*_(1,35)_ = 20.94, *p* < 0.0001, *η*^2^ = 0.37). For ITI response latency (Figure 2C) there was a Group × Trials interaction effect (*F*_(1,35)_ = 6.18, *p* < 0.02, *η*^2^ = 0.15) and a Group main effect (*F*_(1,35)_ = 21.55, *p* < 0.0001, *η*^2^ = 0.38): the latency was longer in CSS than CON mice; whilst it decreased in CSS mice from trials 1–30–51–80, it remained low and constant in CON mice. These group differences were reflected in the CS/ITI reciprocal ratio (Figure 2D), which provided the measure of CS-reward learning. There was a Group × Trials interaction effect (*F*_(1,35)_ = 12.06, *p* < 0.001, *η*^2^ = 0.26): both groups had a ratio value of close to 1 at trials 1–30, with the ratio slightly higher in CSS than CON mice (*p* < 0.02, *η*^2^ = 0.16), and then the ratio increased in CON mice specifically, such that CSS resulted in a relatively low ratio, at trials 51–80 (*p* < 0.03, *η*^2^ = 0.14). In session 2, across all 50 trials CON mice obtained 46 ± 6 pellets and CSS mice obtained 30 ± 16 pellets (*t*_(35)_ = −4.00, *p* < 0.0003).

**Table 1:** Details of mouse body weight and home-cage feeding in the three experiments.

Experiment	Group (*N*)	Baseline BW g (WK)^1^	Daily chow during CSS (g)	% Baseline chow during CSS	Age (Wk)^2^	% BBW during testing	Daily chow during testing (g)	% Baseline chow during testing
Experiment 1	CON (18)	28.4 ± 1.9 (14)	3.5 ± 0.3	99.7% ± 8.0%	18	100.4% ± 1.8%	3.2 ± 0.3	95.1% ± 7.6%
	CSS (19)	27.5 ± 2.5 (14)	4.6 ± 0.6***	122.5% ± 14.3%***	18	101.7% ± 2.3%*	4.0 ± 0.4***	115.1% ± 10.8%***
Experiment 2	Naive (21)	28.5 ± 2.2 (12)			15	97.6% ± 2.1%	3.3 ± 0.3	96.7% ± 7.0%
Experiment 3	CON (10)	27.7 ± 2.0 (12)	3.7 ± 0.3	102.4% ± 7.5%	16	100.6% ± 1.3%	3.4 ± 0.3	96.8% ± 6.1%
	CSS (10)	28.6 ± 1.4 (12)	4.5 ± 0.5***	135.0% ± 8.4%***	16	101.4% ± 1.2%	4.2 ± 0.6***	125.0% ± 12.1%***

^1^Age at assessment of baseline body weight (BBW). ^2^Age at onset of behavioral testing. *p < 0.05, ***p < 0.001, for CSS versus CON in unpaired *t*-test.

In the EVT with a shallow PRS, effortful responding was reduced in CSS mice, as indicated by the lower numbers of operant responses (Figure 2E; *t*_(35)_ = −2.72, *p* < 0.01, *d* = 0.90), and, therefore, pellets obtained (Figure 2F; *p* < 0.01, *d* = 0.90) and lower final ratio attained (Figure 2G; *p* < 0.02, *d* = 0.87).

### Experiment 2

**Figure 3:**
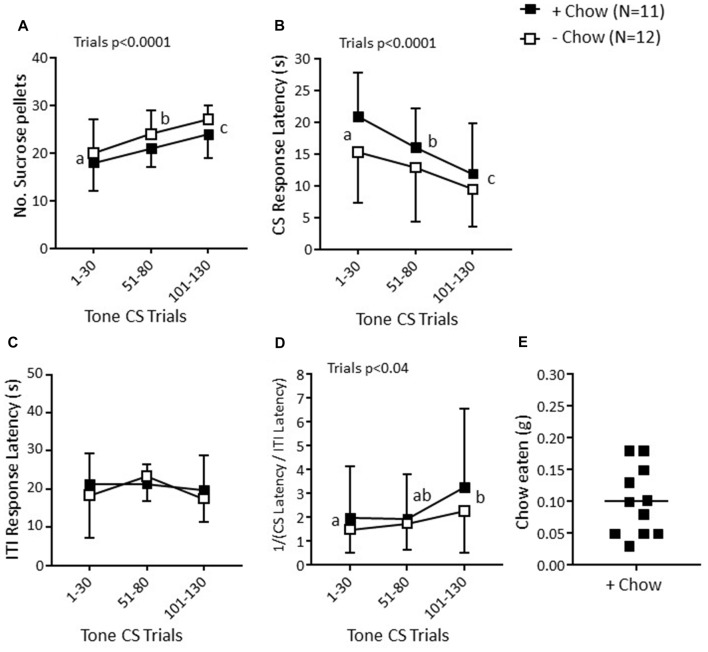
Effects of provision of a chow pellet on behavior in the RST in non-manipulated mice in Experiment 2. Scores are for the first 30 trials per session on three consecutive days. **(A)** Number of sucrose pellets obtained. **(B)** Mean response latency to a tone CS (maximum duration 30 s) associated with pellet availability. **(C)** Mean response latency in the inter-trial intervals (ITI, 50 ± 30 s) between successive CS. **(D)** Mean CS/ITI reciprocal ratio. Values are overall mean ± SD. Significant statistical effects indicated were obtained with mixed factorial ANOVA; trials denoted with different letters were significantly different in *post hoc* Bonferroni tests. **(E)** In the +Chow group, scatter plot and overall mean for the mean weight of chow eaten during the three test sessions.

Details of BW and home cage feeding are given in Table 1. In the RST, mice in the +chow group ate 0.1 ± 0.05 g of chow (Figure 3E). There was no effect of the chow pellet availability in the test chamber on any RST measure (*p* ≥ 0.16, Figures 3A–D). The number of sucrose pellets obtained (Figure 3A) increased consistently across sessions (*F*_(2,42)_ = 22.85, *p* < 0.0001, *η*^2^ = 0.55). CS response latency (Figure 3B) decreased consistently across sessions (*F*_(2,42)_ = 19.04, *p* < 0.0001, *η*^2^ = 0.50), whereas the ITI response latency (Figure 3C) remained constant across sessions (*p* = 0.39). These measures were reflected in the CS/ITI reciprocal ratio (Figure 3D), which was close to 1 at trials 1–30 and then increased significantly (*F*_(2,42)_ = 3.71, *p* < 0.04, *η*^2^ = 0.18) to being higher at trials 101–130 than 1–30 (*p* < 0.03, *η*^2^ = 0.34).

**Figure 4:**
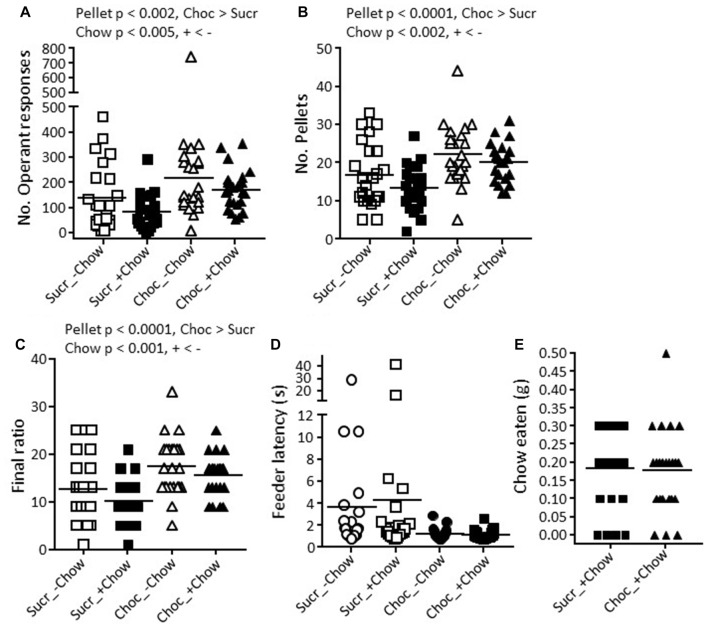
Effects of provision of a chow pellet and of sweet pellet type on behavior in the EVT in non-manipulated mice in Experiment 2. Each mouse was tested under each condition, first with sucrose (Sucr) and then with chocolate (Choc) pellets, and by counterbalancing with respect to whether they were tested first with (+) or without (−) a chow pellet. **(A)** Number of operant responses. **(B)** Number of sucrose pellets earned. **(C)** Final ratio attained. **(D)** Mean latency to collect the pellet from the feeder. Graphs are scatter plots and mean values per group. Significant statistical effects indicated were obtained with 2 × 2 repeated measures ANOVA with within-subject factors of reward and chow pellet. **(E)** In the +Chow conditions, scatter plots and overall means for the weight of chow eaten during the test session.

In the EVT, mice in the +chow groups ate 0.18 ± 0.11 g chow with sucrose pellets and 0.18 ± 0.12 g chow with chocolate pellets (Figure 4E). Despite chocolate pellets (20 mg) being heavier than sucrose pellets (14 mg), the number of operant responses (Figure 4A) was higher with chocolate than with sucrose pellets (Pellet main effect: *F*_(1,22)_ = 12.89, *p* < 0.002, *η*^2^ = 0.37), whilst the presence of chow decreased the number of operant responses (Chow main effect: *F*_(1,22)_ = 9.57, *p* < 0.005, *η*^2^ = 0.30). Accordingly, mice obtained more chocolate than sucrose pellets (Figure 4B) (*F*_(1,22)_ = 23.06, *p* < 0.0001, *η*^2^ = 0.51) and obtained more pellets with −chow than +chow (*F*_(1,22)_ = 12.29, *p* < 0.002, *η*^2^ = 0.36). Mice also attained a higher final ratio (Figure 4C) with chocolate than sucrose pellets (*F*_(1,22)_ = 22.86, *p* < 0.0001, *η*^2^ = 0.51) and with −chow than +chow (*F*_(1,22)_ = 14.29, *p* < 0.001, *η*^2^ = 0.39). There was no significant effect of pellet type (*p* = 0.08) or chow availability (*p* = 0.44) on the latency to retrieve the pellet from the feeder (Figure 4D).

#### Experiment 3

**Figure 5:**
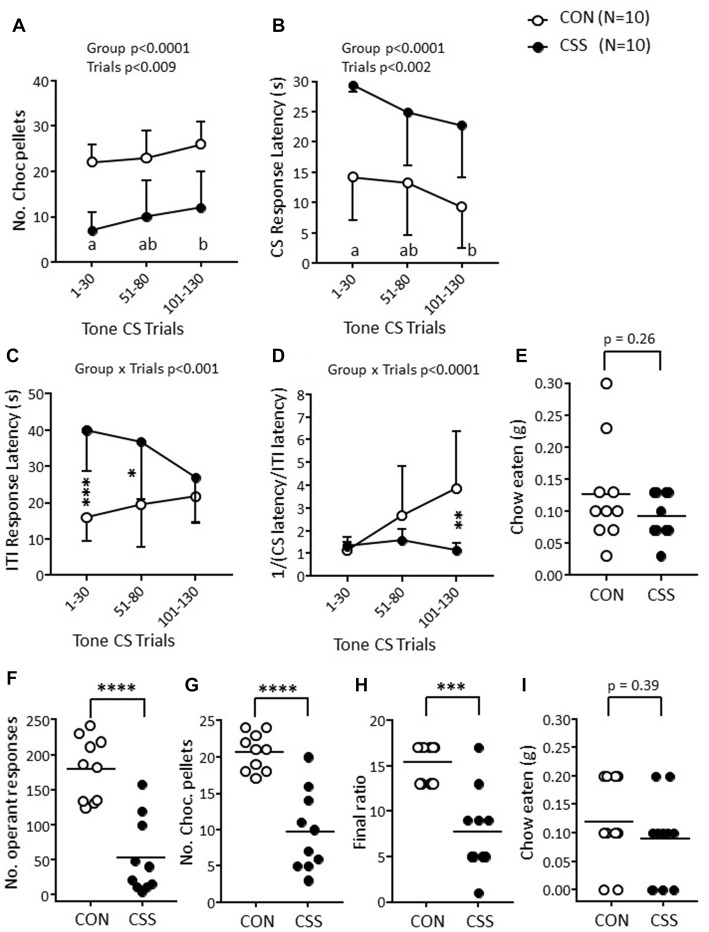
Effects of CSS on days 1–15 on reward-directed behavior in Experiment 3. **(A–D)** Scores for the first 30 trials per session in the RST on days 16–18: **(A)** Number of sucrose pellets obtained. **(B)** Mean response latency to a tone CS (maximum duration 30 s) associated with pellet availability. **(C)** Mean response latency in the inter-trial intervals (ITI, 50 ± 30 s) between successive CS. **(D)** Mean CS/ITI reciprocal ratio. Values are overall mean ± SD. Significant statistical effects indicated were obtained with mixed factorial ANOVA; *p < 0.05, **p < 0.01, ***p < 0.001, in *post hoc* tests of single sessions following Group × Trial interaction. **(E)** Scatter plot and overall mean for the mean weight of chow eaten during the three test session; *p* value is for the unpaired *t*-test. **(F–I)** Scores for the EVT on day 20, using a steep PRS. **(F)** Number of operant responses. **(G)** Number of chocolate pellets earned. **(H)** Final ratio attained. **(I)** Weight of chow eaten during the session. Graphs are scatter plots and mean values per group. Significant statistical effects indicated were obtained with unpaired *t*-tests; ***p < 0.001, ****p < 0.0001.

During the 15-day environmental manipulation period, CON and CSS mice were maintained at 99.8% ± 0.9% and 98.5% ± 1.1% BBW, respectively. This required providing 102% of baseline daily chow in CON mice and 135% thereof in CSS mice (Table 1). During the period of behavioral testing, both groups were again maintained at BBW, and again this required close to the baseline amount of chow in CON mice and 125% thereof in CSS mice (Table 1). In the RST, CSS mice obtained less chocolate pellets (Figure 5A) than CON mice across the three sessions (*F*_(1,18)_ = 37.95, *p* < 0.0001, *η*^2^ = 0.68). Both CON and CSS mice increased the number of pellets obtained across sessions (*F*_(2,36)_ = 5.37, *p* < 0.009, *η*^2^ = 0.23), obtaining more in trials 101–130 than trials 1–30. The CS response latency (Figure 5B) was higher in CSS than CON mice (F(1, 18 = 28.68, *p* < 0.0001, *η*^2^ = 0.61). In CON and CSS mice it decreased consistently across sessions (*F*_(2,36)_ = 7.59, *p* < 0.002, *η*^2^ = 0.30), being lower in trials 101–130 than trials 1–30 (*p* < 0.005, *η*^2^ = 0.47). For ITI response latency (Figure 5C) there was a Group × Trials interaction effect (*F*_(2,36)_ = 8.51, *p* < 0.001, *η*^2^ = 0.32): whereas latency started low and increased in CON mice it started high and decreased in CSS mice; it was higher in CSS than CON mice at trials 1–30 (*p* < 0.0001) and 51–80 (*p* < 0.02) and similar in the two groups in trials 101–130. These group differences were reflected in the CS/ITI reciprocal ratio (Figure 5D), where there was also a Group × Trials interaction effect (*F*_(2,36)_ = 9.92, *p* < 0.0001, *η*^2^ = 0.37): both groups had a ratio close to 1 at trials 1–30, and then the ratio increased in CON mice specifically, so that CSS resulted in a relatively low ratio at trials 101–130 (*p* < 0.003, *η*^2^ = 0.41). Both CON and CSS mice ate an average of 0.1 g chow during each RST test (Figure 5E). In session 3, across all 50 trials, CON mice obtained 39 ± 8 pellets and CSS mice obtained 17 ± 11 pellets (*t*_(18)_ = −5.14, *p* < 0.0001).

In the EVT with a steep PRS, effortful responding was reduced in CSS relative to CON mice, as indicated by the lower numbers of operant responses (Figure 5F; *t*_(18)_ = −5.69, *p* < 0.0001, *d* = 2.54) and, therefore, pellets obtained (Figure 5G; *p* < 0.0001, *d* = 2.56) and lower final ratio attained (Figure 5H; *p* < 0.0002, *d* = 2.13). Both CON and CSS mice ate an average of 0.1 g chow during each EVT test (Figure 5I). In contrast to Experiment 1 where a shallow PRS was used and CON and CSS mice obtained a similar number of pellets per session in the RST and EVT (Figure 2), in this experiment both CON and CSS mice obtained less pellets per session in the steep EVT than in the RST (Figure 5).

**Figure 6:**
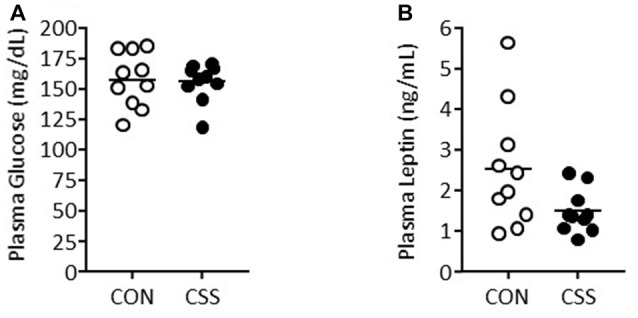
Effect of CSS on days 1–15 on plasma values in Experiment 3: **(A)** Glucose, **(B)** Leptin.

On the day after completion of behavioral testing (day 21), trunk blood samples were collected. There was no effect of CSS on plasma glucose levels (Figure 6A): CON mice had 157 ± 25 and CSS mice 156 ± 16 mg/dL (*p* = 0.84). There was no effect of CSS on plasma levels of the appetite-suppressant adipokine leptin (Figure 6B): CON mice had 2.54 ± 1.69 and CSS mice 1.50 ± 0.54 ng/mL (*t*_(18)_ = −2.07, *p* = 0.053, *d* = 0.83).

### Discussion

Animal models of stress-induced deficits in reward processing that have analogy with human reward psychopathologies are essential for detailed study of the underlying pathophysiology. Here, we report in mice that CSS leads to deficits in behavioral tests for the dimensions of reward salience and effort valuation relative to gustatory stimuli, and identify some test variables that are important in determining the robustness of these CSS effects.

The RST was used to determine whether CSS would lead to a deficit in one or both of appetitive responding for sweet-tasting reward under minimally effortful conditions and learning the association between a CS and reward availability. In Experiments 1 and 3, CSS mice obtained less sweet pellets than CON mice, due to a longer response latency during the CS. This deficit was consistent across sessions, although both CON and CSS mice obtained more rewards as they progressed through trials/sessions. In contrast to CON mice, there was no evidence for acquisition of the tone-reward association in CSS mice, as demonstrated by their CS/ITI reciprocal ratio remaining at a value close to 1. Of course, these two effects of CSS are inter-dependent, with reduced interest in reward leading to reduced appetitive responding and reduced exposure to the predictive associations of tone-reward and ITI no tone-no reward. Nonetheless, the combination of increased number of pellets obtained across sessions and absence of Pavlovian learning suggests that a deficit in reward learning does contribute to the CSS effect in this test. Reductions in reward salience, learning and expectancy are also implicated in the deficits in development of response bias in a signal-reward detection test as observed in human subjects scoring high on the Beck Depression Inventory (Pizzagalli et al., 2005) and in rats that had experienced 3 days of social stress (Der-Avakian et al., 2017). With regards to the neurobiology underlying the deficits of CSS mice in the RST, the evidence suggests involvement of the basolateral nucleus of the amygdala. In mice exposed to a similar tone-sucrose test, glutamatergic neurons in the basolateral amygdala that projected to the nucleus accumbens displayed an increase in synaptic strength, based on the AMPA receptor/NMDA receptor ratio. This was determined by measuring receptor-specific excitatory post-synaptic current amplitude using whole-cell patch-clamp recording in neurons obtained from tone-sucrose conditioned mice and unconditioned control mice (Namburi et al., 2015). Therefore, it will be important to investigate the effects of CSS on these “reward neurons” projecting from basolateral nucleus to nucleus accumbens (Pryce, 2018).

Comparing the two CSS-RST experiments, the number of pellets obtained was higher and the CS and ITI response latencies shorter in Experiment 1 compared with 3. This was despite Experiment 3 being conducted with chocolate pellets, which were more of an incentive than sucrose pellets, at least according to the direct comparison in the EVT in Experiment 2. Experiment 2 also demonstrated that the presence of normal chow did not impact significantly on sucrose pellets earned in the RST, so this cannot account for the overall reduction in reward salience in Experiment 3 vs. 1. Otherwise, the most notable difference between the two experiments was that the mice in Experiment 1 underwent a shift in the light-dark cycle at age 5 weeks (see “Animals and Housing” section). Given the sensitive inter-relationships between circadian rhythmicity, its disruption, levels of appetite hormones and glucose metabolism (Kim et al., 2015), the circadian shift might have caused a chronic increase in sucrose sensitivity. Whilst this remains to be clarified, it is encouraging that the CSS-RST model is sufficiently robust to accommodate baseline differences in reward salience.

The EVT was used to determine whether CSS would lead to a deficit in appetitive responding for sweet-tasting reward under increasingly effortful conditions. In Experiments 1 and 3, CSS mice obtained less sweet pellets than CON mice due to reduced operant responding. The session duration was always maintained at 45 min, rather than using a break point if there was no operant or feeder response within a certain period; therefore, the overall rate of responding was reduced in CSS mice. Compared with controls, depressed patients made less high-effort/high-reward choices relative to low-effort/low-reward choices (Treadway et al., 2012). Behavior in high-effort/high-reward tests is clearly dependent on reward salience, which the RST had already identified as being reduced in CSS mice. Of particular interest in the EVT is whether there is an additional CSS effect on effort valuation. In Experiment 1, which used a shallow PRS in the absence of a low-effort/low-reward alternative, CON and CSS mice obtained as many sucrose pellets in the EVT as they had in the RST. This is consistent with the EVT not being sufficiently challenging to impact on effort reward valuation and, perhaps related to this, not identifying an increase in effort discounting in CSS mice. Experiment 3 used a steep PRS for responding for chocolate pellets and also provided a low-effort/low-reward alternative. Under these conditions, both CON and CSS mice obtained fewer chocolate pellets in the EVT than they had in the RST. These data are consistent with this EVT being sufficiently challenging to impact on effort valuation of reward. Furthermore, the decrease in operant responses was particularly marked in CSS mice relative to CON mice (vis effect size >2), consistent with CSS increasing effort discounting in addition to the decrease in reward salience under low-effort conditions.

In the EVT and other effort-based tests, the interval or delay between onset of responding and obtaining of reinforcement requires a neurobiological system that maintains motivation. In humans, using a monetary incentive delay task conducted with fMRI, in the interval between stimulus response and reward feedback subjects exhibit increased activity in the ventral striatum/nucleus accumbens and, relative to controls, depressed and schizophrenic patients exhibit lower activation (Arrondo et al., 2015), with the magnitude of activation being inversely correlated with the Beck Depression Inventory score (Hagele et al., 2015). Accordingly, the nucleus accumbens and the dopamine inputs it receives from neurons in the ventral tegmental area could be a major pathway in the regulation of effortful reward-directed behavior. CSS leads to reduced dopamine turnover in the nucleus accumbens and reduces its sensitivity to dopamine release (Bergamini et al., 2018). Furthermore, the effects of CSS in the EVT are analogous to those obtained with pharmacological (6-hydroxydopamine) reduction of dopamine levels in the nucleus accumbens (Bergamini et al., 2016b). Therefore, the reward-sensitive basolateral amygdala glutamate neurons could be a major regulatory region for reward salience, and the nucleus accumbens, integrating inputs from these amygdala glutamate neurons with those from dopamine neurons projecting from the ventral tegmental area, could be a major regulatory region for effort valuation of reward stimuli. Given that the mice had received multiple exposures to the test environment during training, it is unlikely that increased aversive responding by CSS mice to this environment contributed to the effects observed in the EVT or RST.

The observed effects of CSS on reward-directed behavior co-occur with peripheral changes relating to feeding, energy metabolism and appetite regulation. These include increased food intake to maintain BW and a decrease in the appetite-supressing adipokine leptin (Bergamini et al., 2016a). As in previous studies of CSS effects on reward-directed behavior (Bergamini et al., 2016a, 2018), we provided CSS and CON mice with sufficient daily food to maintain them at BBW during CSS and subsequent behavioral testing. This required provision of CSS mice with 20%–30% more food than CON mice. On days of behavioral testing, food was provided 2–3 h after testing and was consumed, by all mice, several hours prior to behavioral testing on the following day. With regards to blood parameters likely to impact on test behavior, in Experiment 3 on the day after completion of behavioral testing, blood samples were collected for determination of plasma glucose and leptin values. Plasma glucose was at typical levels (Togashi et al., 2016) and similar in both CON and CSS mice, indicating that the additional food provision to CSS mice did not elevate circulating glucose. Despite the additional food received, plasma leptin levels still tended to be reduced in CSS relative to CON mice. In free-feeding mice, plasma leptin is markedly reduced in CSS compared with CON mice (Bergamini et al., 2016a), and the major difference between the present study and this previous finding was the lower leptin levels in CON mice, in line with the food restriction used here to maintain 100% BBW. Nonetheless, the lower average plasma leptin in CSS mice suggests that its appetite-suppressant effects would also be lower in CSS than CON mice. Therefore, with respect to both glucose and leptin levels, there was no evidence that the CSS effects of reduced gustatory-reward salience and effort valuation were attributable to changes in chow intake, energy metabolism and appetite regulation, but rather reflected reduced reward sensitivity. The CSS effects on energy metabolism are nonetheless interesting given that: a common symptom of melancholic depression is weight loss, with reduced interest extending to food, much of which will be sweet tasting (DSM-5, 2013); depression and energy metabolism disorders such as type-2 diabetes are highly co-morbid (Knol et al., 2006); and altered energy metabolism, at least in the brain, is well-described for depression, including increased energy uptake by the amygdala, anterior cingulate cortex and frontal cortex (Price and Drevets, 2010; Harper et al., 2017; Scifo et al., 2018). Furthermore, with respect to leptin specifically, depression is associated with low levels of this adipokine hormone and pharmacological studies indicate that leptin has antidepressant-like efficacy (Lu, 2007; Caron et al., 2018).

This study has demonstrated that CSS leads to reduced gustatory reward salience, learning and expectancy under low-effort conditions and an additional reduction in reward valuation under high effort conditions, in mice. Evidence has also been obtained for the behavioral test conditions that yield the most robust CSS effects, including the use of highly flavored food stimuli, provision of low-effort/low-reward normal diet, and a steep PRS. Importantly, the reward dimensions for which stress-induced deficits have been demonstrated in this study are back-translated from deficits in reward processing described for stress-related neuropsychiatric disorders, most notably depression but also negative symptoms in schizophrenia. It would represent an over-interpretation to attempt to equate these effects to mild, moderate or severe depression. What can be stated is that robust and reproducible effects are obtained, such that the model can be applied for the study of pharmacological reversal of CSS effects. Accordingly, the CSS-RST and CSS-EVT pathology models can now be utilized to increase understanding of the pathophysiologies underlying these reward pathologies and their treatment.

### Author Contributions

DK and GB conducted the experiments and data analysis and wrote the manuscript. HS conducted the experiments and data analysis. ES wrote the manuscript. BH and CP designed the study and wrote the manuscript.

### Conflict of Interest Statement

BH is an employee of Boehringer Ingelheim Pharma GmbH & Co. KG, Germany. The research presented in this article was partly funded by a research collaboration between Boehringer Ingelheim Pharma GmbH & Co. KG, Germany and PLaTRAD, University of Zurich, Switzerland. The remaining authors declare that the research was conducted in the absence of any commercial or financial relationships that could be construed as a potential conflict of interest.

## Footnotes

^1^https://www.nimh.nih.gov/research-priorities/rdoc/index.shtml
